# Supplementary material for: Advancing nonadiabatic molecular dynamics simulations in solids with E(3) equivariant deep neural hamiltonians
Source: Nat Commun. 2025 Feb 27;16:2033. doi: 10.1038/s41467-025-57328-1 (PMC11868637; doi:10.1038/s41467-025-57328-1)
Supplement: Supplementary file 1 — Supplementary Information [file 41467_2025_57328_MOESM1_ESM.pdf]

Supplementary Information for: Advancing Nonadiabatic Molecular Dynamics Simulations in Solids with E(3) Equivariant Deep Neural Hamiltonians

Changwei Zhang[1†], Yang Zhong[1†], Zhi-Guo Tao[1], Xinming Qin[2], Honghui Shang[2], Zhenggang Lan[3], Oleg V. Prezhdo[4], Xin-Gao Gong[1], Weibin Chu[1*], Hongjun Xiang[1*]

[1]Key Laboratory of Computational Physical Sciences (Ministry of Education), Institute of Computational Physical Sciences, State Key Laboratory of Surface Physics, and Department of Physics, Fudan University, Shanghai, 200433, China.
[2]Key Laboratory of Precision and Intelligent Chemistry, Hefei National Research Center for Physical Sciences at the Microscale, University of Science and Technology of China, Hefei, Anhui, 230026, China.
[3]SCNU Environmental Research Institute, Guangdong Provincial Key Laboratory of Chemical Pollution and Environmental Safety & MOE Key Laboratory of Environmental Theoretical Chemistry, South China Normal University, Guangzhou, Guangdong, 510006, China.
[4]Department of Chemistry and Department of Physics & Astronomy, University of Southern California, Los Angeles California, 90089, United States.

*Corresponding author(s). E-mail(s): wbchu@fudan.edu.cn; hxiang@fudan.edu.cn;
†These authors contributed equally to this work.

1 Supplementary benchmark of N^2AMD on TiO_2 and GaAs

In this section, we present the additional benchmark of N^2AMD on TiO_2 and GaAs. Similar to the procedure for TiO_2 in the manuscript, the prediction capability of N^2AMD on Hamiltonian matrix elements (Supplementary Fig. 1a), KS orbital energies (Supplementary Fig. 1b) and band structures (Supplementary Fig. 1c) are carefully examined. All the benchmarks consistently yield excellent results compared with DFT calculations. The MAEs of Hamiltonian matrices and KS orbital energies are 0.16meV and 4.8meV respectively.

We also presented the evolution of real-time KS eigenvalues of VBM and CBM along a 100 fs MD trajectory for both TiO_2 and GaAs in Supplementary Fig. 2a-b. The MAEs of eigenvalues for both systems are 2.4meV and 12.0meV respectively. Supplementary Fig. 2c shows the non-adiabatic couplings (NACs) between the orbtial pairs (VBM-2, VBM-1), (VBM-2, VBM) and (VBM-1, VBM) of GaAs. Although the NACs have significantly sharp peaks due to the near degeneracy of these three bands, N^2AMD still perfectly fit DFT calculations with an MAE of 0.78meV. Further, we provide a scatter plot of benchmark results for key quantities of TiO_2 in Supplementary Fig. 2d-e. Considering the difference in the energy scales for orbital energies (eV) and NACs (meV), the predicted orbital energies and NACs fit DFT calculations with satisfying accuracy.

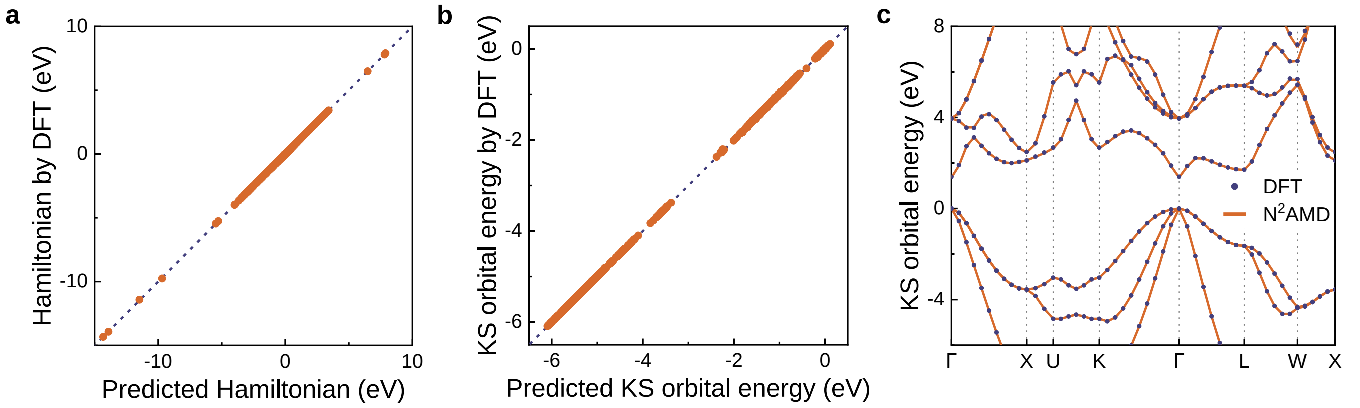

Supplementary Figure 1 Benchmarking Neural-Network Non-Adiabatic Molecular Dynamics (N^2AMD) on GaAs. (**a-c**) Comparison of N^2AMD predicted and density functional theory (DFT) calculated (**a**) Hamiltonian matrices, (**b**) Kohn-Sham (KS) orbital energies, and (**c**) band structures. Source data are provided as a Source Data file.

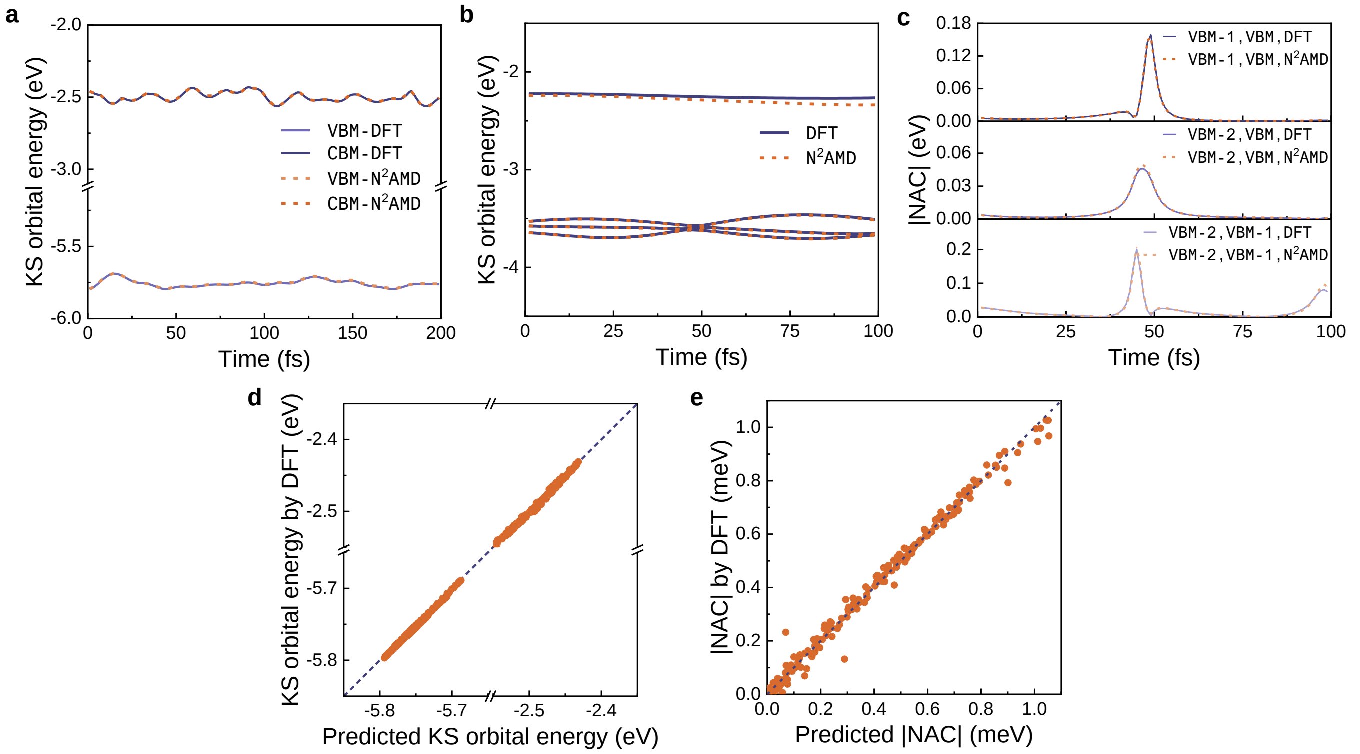

Supplementary Figure 2 Additional benchmark of Neural-Network Non-Adiabatic Molecular Dynamics (N^2AMD) on key quantities in nonadiabatic (NA) molecular dynamics (MD). (**a-b**) Real-time Kohn-Sham (KS) eigenvalues of valence band maximum (VBM) and conduction band minimum (CBM) for (**a**) TiO_2 and (**b**) GaAs along a short MD trajectory. (**c**) Time-dependent absolute values of nonadiabatic couplings (NACs) between the three highest valence bands of GaAs. (**d-e**) Scatter plot of benchmark result for KS orbital energies and NACs of TiO_2. Source data are provided as a Source Data file.

2 Benchmark of N^{2}AMD on wavefunction prediction capability

The prediction capabilities of N^{2}AMD for Kohn-Sham orbital energies and band structures are demonstrated in the manuscript. Here, we present the benchmark results on Kohn-Sham wavefunctions. Given the challenges posed by the random phase of Bloch wavefunctions, we have opted to compare the real-space wavefunctions as predicted by N^{2}AMD and computed via DFT. As shown in Supplementary Fig. 3, the real-space wavefunctions for both the valence band maximum (VBM) and conduction band minimum (CBM) of TiO_2, as predicted by N^{2}AMD, perfectly match those calculated by DFT. This excellent performance in wavefunction prediction is particularly beneficial for NAC predictions, as NACs are derived by evaluating the overlap between two instantaneous wavefunctions.

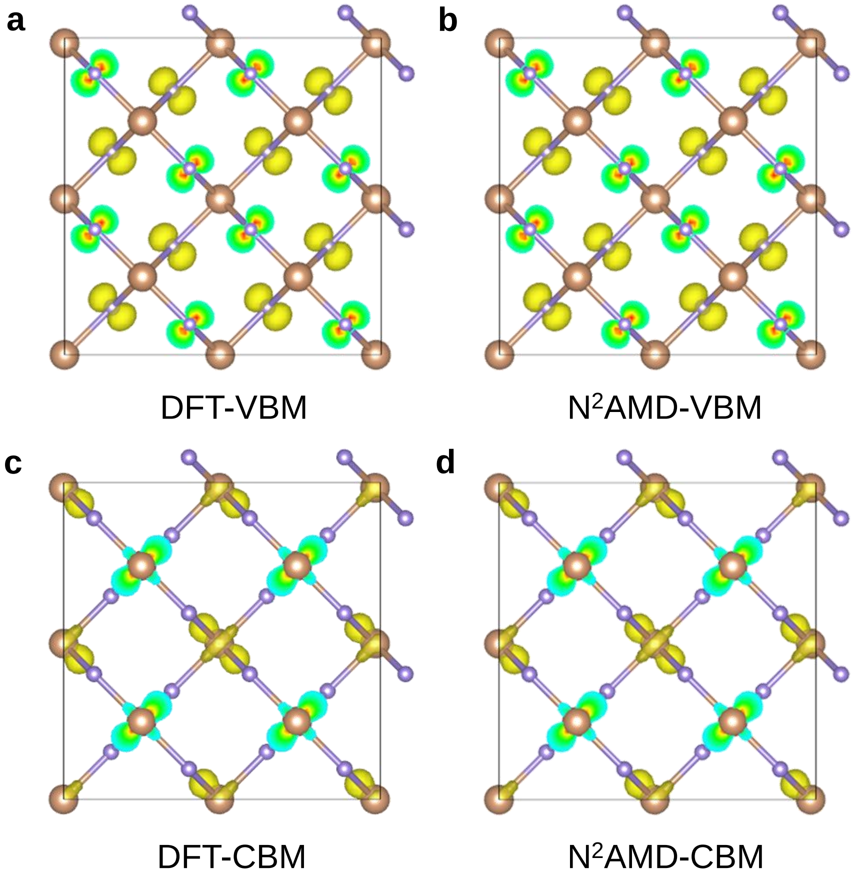

Supplementary Figure 3 Benchmark of Neural-Network Non-Adiabatic Molecular Dynamics (N^{2}AMD) on wavefunction prediction. (**a**) and (**c**) The real-space wavefunction of valence band maximum (VBM) and conduction band minimum (CBM) calculated with DFT. (**b**) and (**d**) The wavefunction of VBM and CBM predicted by N^{2}AMD.

3 Comparative study with conventional methods

To thoroughly evaluate the performance of our workflow, we compare the accuracy and generalizability of N^2AMD with conventional methods that directly predict NACs and Kohn-Sham orbital energies, which include a KRR model from our previous work[1] and an MLP model using the same feature as the KRR model. Bilayer MoS_2 is utilized as the benchmark system. All three models are trained on 40 structures, uniformly sampled from the interpolation zone (yellow background in Supplementary Fig. 4), and tested on the 1000 structures along the NVE trajectory at 300K.

As shown in Supplementary Fig. 4, N^2AMD outperforms the other two methods in predicting both KS orbital energies and NACs. The MAEs for the VBM, CBM energies, and NACs are 1.5 meV, 2.2 meV, and 0.038 meV, respectively (Supplementary Table 1), which are four to ten times smaller than those obtained from the conventional methods. Furthermore, the conventional method suffers from significantly poor performance in the extrapolation zone, as also noted in the previous study. In contrast, N^2AMD predicts the instantaneous Hamiltonian and calculates key quantities in NAMD afterward, ensuring excellent predictive capability in both the interpolation and extrapolation zones. Additionally, conventional methods exhibit zero transferability to systems of different sizes or chemical compositions, as their trainable parameters are only applicable to a fixed feature dimension.

Supplementary Table 1 The mean absolute error (MAE) of key quantities in nonadiabatic molecular dynamics (NAMD) predicted by kernel ridge regression (KRR), multilayer perception (MLP) and Neural-Network NAMD (N^2AMD). Only data from the interpolation zone is used to calculate the MAE values. The hyperparameters of KRR model are tuned to $\xi = 1, \eta = 0.1, R_C = 5.5\text{Å}, \alpha = 0.0001$. Five hidden layers with the number of neurons (256, 256, 256, 64, 16) are used in the MLP model. The best model with the least loss in the training dataset is used for inference. N^2AMD achieves the best performance on both orbital energies (including valance band maximum (VBM) and conduction band minimum (CBM)) and nonadiabatic couplings (NACs).

Model	MAE E_{VBM} (meV)	MAE E_{CBM} (meV)	MAE NAC (meV)
KRR	24.2	19.8	0.125
MLP	12.3	11.3	0.157
N^2AMD	1.5	2.2	0.038

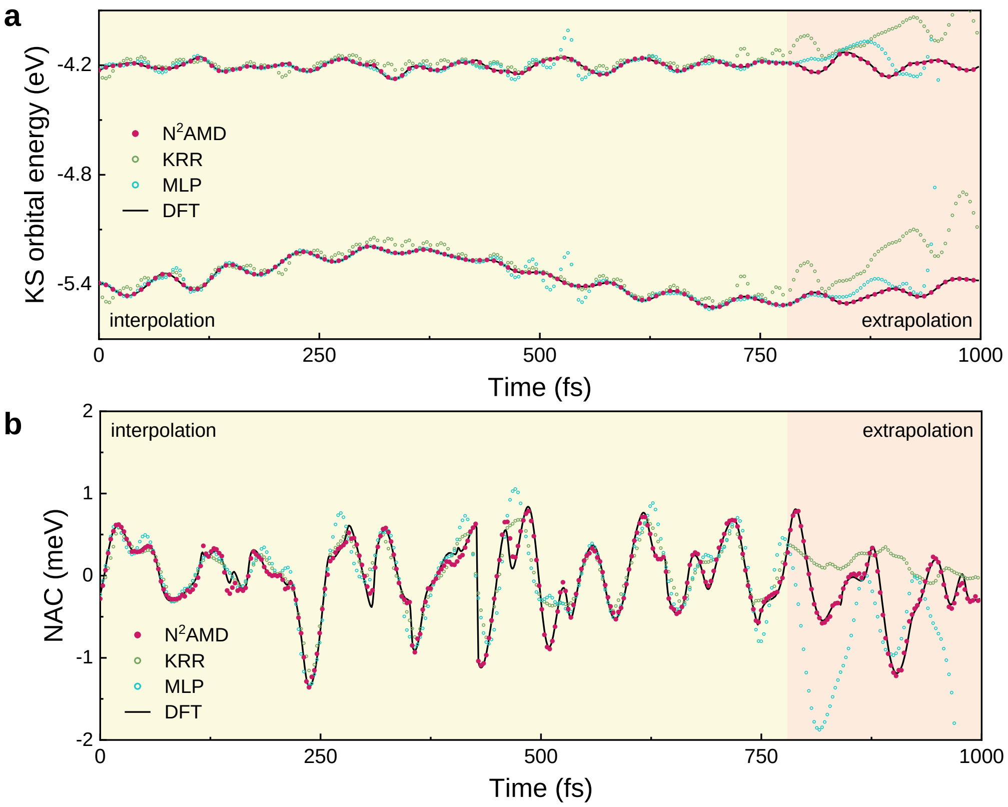

Supplementary Figure 4 The performance of kernel ridge regression (KRR), multilayer perception (MLP) and Neural-Network Non-Adiabatic Molecular Dynamics (N^2AMD) on both (**a**) Kohn-Sham (KS) orbital energies and (**b**) nonadiabatic couplings (NACs). The entire trajectory is divided into an interpolation zone (yellow background) and an extrapolation zone (orange background), according to whether the training datasets lie in the area. Source data are provided as a Source Data file.

4 Transferability of N^2AMD on twist-angle MoS_2 bilayer and silicon nanotube

In this section, we present the time evolution results of KS eigenvalues and NACs of the twist-angle MoS_2 bilayer and silicon nanotube. As demonstrated in Supplementary Fig. 5, for both systems, the KS orbital energies of VBM and CBM, and absolute values of NACs predicted by N^2AMD perfectly reproduce the results of DFT, except minor acceptable differences at the peaks of the NACs. For the twist-angle MoS_2 bilayer, the MAE of KS eigenvalues and absolute NAC values are 5.1meV and 0.027meV respectively. For the silicon nanotube, the VBM energy at 0 fs is set to zero, and the canonically averaged CBM energy is aligned with a shift of 11.9 meV. After the alignment, the MAE of KS eigenvalues and absolute NAC values are 0.91 meV and 0.11 meV.

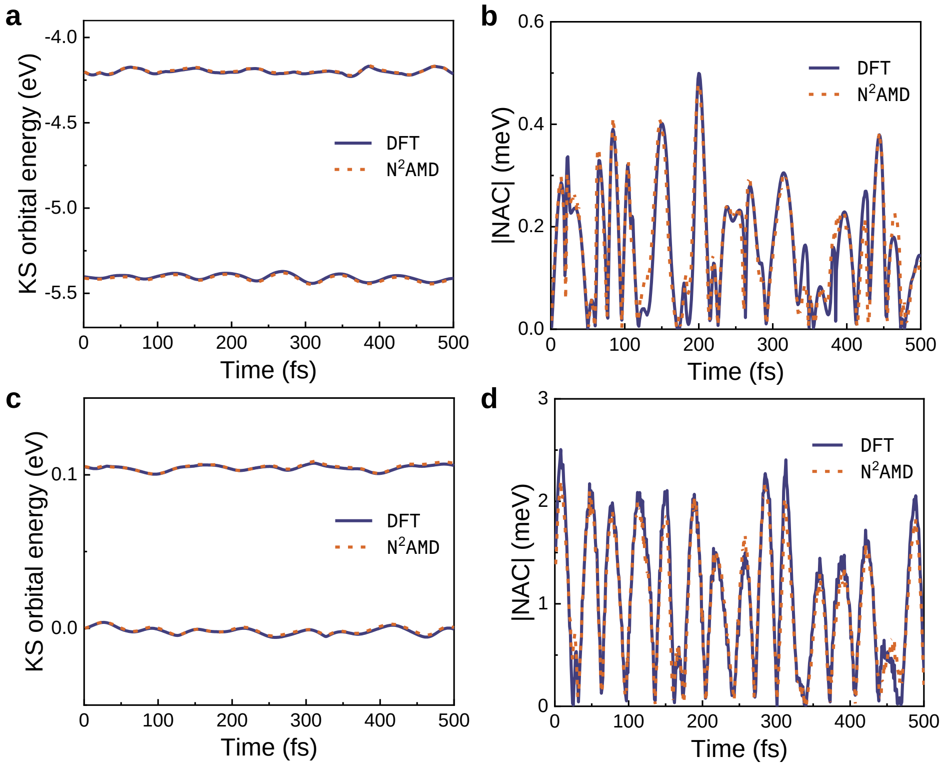

Supplementary Figure 5 Generalization capability of Neural-Network Non-Adiabatic Molecular Dynamics (N^2AMD) on Kohn-Sham (KS) orbital energy and nonadiabatic coupling (NAC) predictions. (**a-b**) Twist-angle MoS_2 bilayer. (**a**) Evolution of valence band maximum (VBM) and conduction band minimum (CBM) KS orbital energies, and (**b**) evolution of absolute NAC values, calculated by density functional theory (DFT) and N^2AMD. (**c-d**) Silicon nanotube. (**c**) Evolution of VBM and CBM relative energies, and (**d**) evolution of absolute NAC values, calculated by DFT and N^2AMD. Source data are provided as a Source Data file.

5 Localization in large-scale NAMD simulation

To determine the origin of the prolonged recombination lifetime and narrowed bandgap found in the large-scale NAMD simulation, we further investigate the wavefunction of frontier orbitals, by calculating wavefunction distributions of structures randomly selected from the MD trajectory. It is shown that the real-space wavefunction of CBM is considerably localized even in PBE calculations (Supplementary Fig. 6b) while the VBM wavefunction still exhibits delocalization properties (Supplementary Fig. 6a). This localization of the CBM wavefunction suggests that the markedly decreased NACs, resulting from this localization, are a primary factor extending the recombination lifetime in a large-scale simulation cell. This observation aligns with our NAC results. We perform the same calculation for the HSE06 functional and find a similar localization phenomenon of CBM (Supplementary Fig. 6d). However, even though PBE is already capable of capturing localization features in CBM, HSE06 gives more localized results for both VBM and CBM (Supplementary Fig. 6c and 6d), proving that efficient hybrid functional simulation implemented by N^2AMD is crucial in accurately investigating these complicated phenomena.

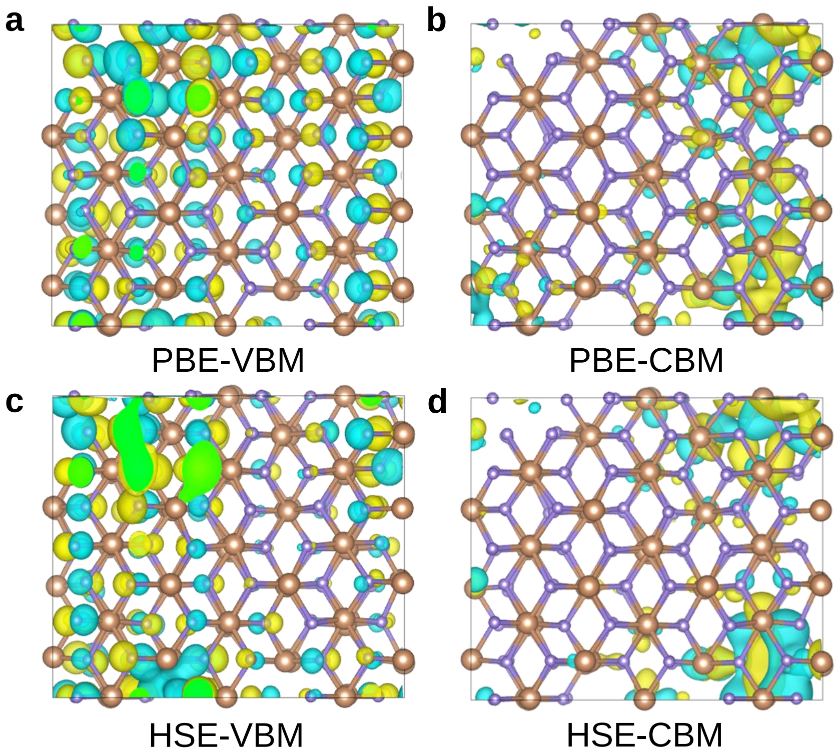

Supplementary Figure 6 Real-space wavefunctions of large-scale simulation cell. (**a-b**) Real-space wavefunctions of (**a**) valance band maximum (VBM) and (**b**) conduction band minimum (CBM) calculated by PBE via density functional theory (DFT). The atomic structure is randomly selected from the MD trajectory. (**c-d**) Real-space wavefunctions of (**c**) VBM and (**d**) CBM calculated by HSE06 via eural-Network Non-Adiabatic Molecular Dynamics (N^2AMD). The atomic structure is picked from the HSE MD trajectory so that it is different from the structure in (**a**) and (**b**).

6 Defect state properties calculated by different exchange-correlation functionals

This section presents the properties of positively charged oxygen vacancy (V_O^+) in rutile TiO_2 computed by PBE and HSE06 functionals. In both calculations, the atomic structure is fully relaxed using the corresponding exchange-correlation functional until the maximum ionic force on each of the atoms is less than 0.01 eV/Å. As shown in Supplementary Fig. 7, the PBE functional suggests that the position of the vacancy state should be extremely close to fundamental CBM, resulting in a shallow defect level. While the HSE06 functional predicts it to be a deep defect state in the bandgap. The energy difference between the defect state and fundamental CBM is 1.23 eV. The state is predominantly localized around two titanium atoms adjacent to the oxygen vacancy, manifesting as d-wave orbitals. This observation is consistent with previous research [2].

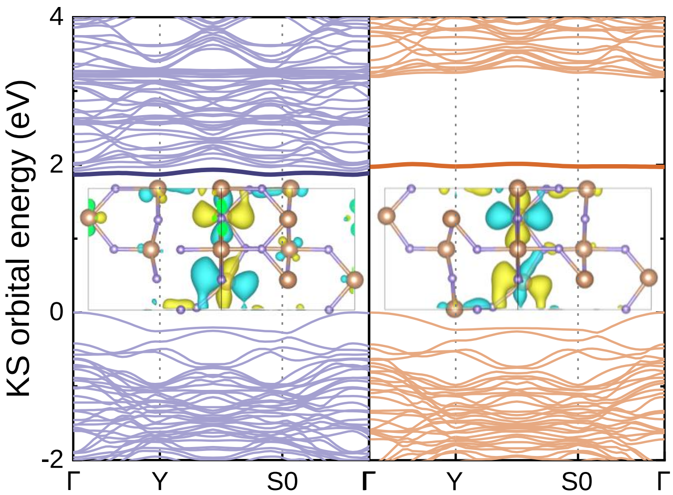

Supplementary Figure 7 PBE (left panel) and HSE06 (right panel) calculated Kohn-Sham (KS) band structures of positive charged oxygen vacancy (V_O^+) TiO_2. In both panels, the bold line near 2 eV represents the vacancy state. The real-space wavefunctions of this vacancy state are displayed in the subfigures within each panel. Source data are provided as a Source Data file.

7 Computational costs of N^2AMD on nonadiabatic coupling vectors

This section presents the detailed computational cost of our model on nonadiabatic coupling vectors (NACVs). Using bilayer MoS_2 as an example, in the training process, the time required for Hamiltonian training is approximately 3 minutes and 13 seconds per epoch on a single NVIDIA A800 GPU. For typical semiconductors, around 500 epochs are sufficient. The time for fine-tuning orbital energies is 19 minutes and 13 seconds per epoch on 4 NVIDIA A800 GPUs. This stage is more time-consuming due to the diagonalization of the Hamiltonian during training; however, only about 20 to 50 epochs are necessary for this step.

In contrast to the training phase, the prediction of the Hamiltonian takes only a few seconds. For the computational cost of NACVs with ML Hamiltonian employing finite difference, we benchmarked our model using 50 rutile TiO_2 structures with 6 atoms each. The computational cost of N^2AMD is 2.36×10^4 core·seconds, compared to 5.84×10^7 core·seconds for DFT calculations.

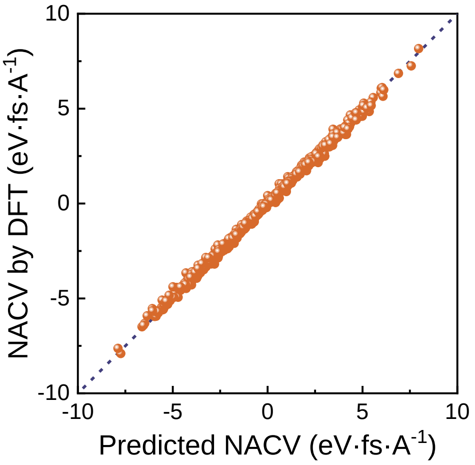

Supplementary Figure 8 Comparison of Neural-Network Non-Adiabatic Molecular Dynamics (N^2AMD) predicted and density functional theory (DFT) calculated nonadiabatic coupling vectors (NACVs). The three components in the 3D space are reduced to 1D data for plotting. Source data are provided as a Source Data file.

8 Details of decoherence induced surface hopping

In the carrier dynamics of recombination, the decoherence effect has to be carefully considered. A scheme like fewest switches surface hooping (FSSH)[3] suffers from severe overcoherence problems, which will lead to an overestimated recombination rate. Decoherence induced surface hopping (DISH)[4] introduces the decoherence effect to the dynamics by assuming that the quantum transitions are triggered barely at the time of decoherence. The decoherence time $\tau_i(t)$ can vary for different states, which is defined as:

$$\frac{1}{\tau_i(t)} = \sum_{j=1, j\neq i}^{N} |c_j(t)|^2 r_{ij} \tag{S1}$$

where subscripts i and j are state indices, and r_{ij} are decoherence rates between pairs of states. Numerous approaches have been developed for calculating the decoherence rate. In this work, r_{ij} is precalculated using the decoherence function:

$$D_{ij} = \exp\left(-\frac{1}{\hbar^2}\int_0^t d\tau_2 \int_0^{\tau_2} d\tau_1 \left\langle \delta E_{ij}(T)\delta E_{ij}(\tau_1 - T)\right\rangle_T\right) \tag{S2}$$

where $\langle\cdot\rangle_T$ denotes canonical averaging, and $\delta E_{ij}(t) = \Delta E_{ij}(t) - \langle \Delta E_{ij}(t)\rangle_t$ is the fluctuation of the energy gap between two states. By fitting the decoherence function with a Gaussian, the decoherence rate r_{ij} can be found as the inverse of the Gaussian smearing.

In the DISH algorithm, decoherence events only take place when the time interval between two events reaches decoherence time $t_i(t) > \tau_i(t)$. At the decoherence event, the trajectory attempts to hop to state i with probability $|c_i(t)|^2$, and the wavefunction collapses to state i simultaneously. Otherwise, the hop is rejected, and the wavefunction projects out by setting $c_i(t) = 0$ and imposing a renormalization procedure. Under the CPA, the probability of the hops upward in energy scope is scaled by the Boltzmann factor $\exp\left(-\Delta E/k_B T\right), \Delta E > 0$.

Since the stochastic nature of the trajectory hopping methodology, hundreds of trajectories have to be simulated to achieve a reasonable result. The time-dependent population of the state is found as:

$$p_i^{\mathrm{SH}} = \frac{N_i(t)}{N_{\mathrm{traj}}} \tag{S3}$$

where $N_i(t)$ is the number of SH trajectories in state i at time t and N_{traj} is the total number of trajectories in the ensemble.

References

[1] Wu, Y., Prezhdo, N. & Chu, W. Increasing Efficiency of Nonadiabatic Molecular Dynamics by Hamiltonian Interpolation with Kernel Ridge Regression. *The Journal of Physical Chemistry A* **125**, 9191–9200 (2021).

[2] Janotti, A. *et al.* Hybrid functional studies of the oxygen vacancy in TiO 2. *Physical Review B* **81**, 085212 (2010).

[3] Tully, J. C. Molecular dynamics with electronic transitions. *The Journal of Chemical Physics* **93**, 1061–1071 (1990).

[4] Jaeger, H. M., Fischer, S. & Prezhdo, O. V. Decoherence-induced surface hopping. *The Journal of Chemical Physics* **137**, 22A545 (2012).
